# Genetic and Genomic Analysis of a Fat Mass Trait with Complex Inheritance Reveals Marked Sex Specificity

**DOI:** 10.1371/journal.pgen.0020015

**Published:** 2006-02-03

**Authors:** Susanna Wang, Nadir Yehya, Eric E Schadt, Hui Wang, Thomas A Drake, Aldons J Lusis

**Affiliations:** 1 Department of Human Genetics, David Geffen School of Medicine, University of California Los Angeles, Los Angeles, California, United States of America; 2 Rosetta Inpharmatics, Seattle, Washington, United States of America; 3 Department of Statistics, College of Letters and Science, University of California Los Angeles, Los Angeles, California, United States of America; 4 Department of Pathology and Laboratory Medicine, David Geffen School of Medicine, University of California Los Angeles, Los Angeles, California, United States of America; 5 Department of Medicine, Division of Cardiology, David Geffen School of Medicine, University of California Los Angeles, Los Angeles, California, United States of America; 6 Department of Microbiology, Immunology, and Molecular Genetics, College of Letters and Science, University of California Los Angeles, Los Angeles, California, United States of America; 7 Molecular Biology Institute, University of California Los Angeles, Los Angeles, California, United States of America; North Carolina State University, United States of America

## Abstract

The integration of expression profiling with linkage analysis has increasingly been used to identify genes underlying complex phenotypes. The effects of gender on the regulation of many physiological traits are well documented; however, “genetical genomic” analyses have not yet addressed the degree to which their conclusions are affected by sex. We constructed and densely genotyped a large F2 intercross derived from the inbred mouse strains C57BL/6J and C3H/HeJ on an apolipoprotein E null (ApoE^−/−^) background. This BXH.ApoE^−/−^ population recapitulates several “metabolic syndrome” phenotypes. The cross consists of 334 animals of both sexes, allowing us to specifically test for the dependence of linkage on sex. We detected several thousand liver gene expression quantitative trait loci, a significant proportion of which are sex-biased. We used these analyses to dissect the genetics of gonadal fat mass, a complex trait with sex-specific regulation. We present evidence for a remarkably high degree of sex-dependence on both the *cis* and *trans* regulation of gene expression. We demonstrate how these analyses can be applied to the study of the genetics underlying gonadal fat mass, a complex trait showing significantly female-biased heritability. These data have implications on the potential effects of sex on the genetic regulation of other complex traits.

## Synopsis

Although their genomes are nearly identical, the males and females of a species exhibit striking differences in many traits, including complex traits such as obesity. This study combines genetic and genomic tools to identify in parallel quantitative trait loci (QTLs) for a measure of gonadal fat mass and for expression of transcripts in the liver. The results are used to explore the relationship between genetic variation, sexual differentiation, and obesity in the mouse model. Using over 300 intercross progeny of two inbred mouse strains, five loci in the genome were found to be highly correlated with abdominal fat mass. Four of the five loci exhibited opposite effects on obesity in the two sexes, a phenomenon known as sexual antagonism. To identify candidate genes that may be involved in obesity through their expression in the liver, global gene expression analysis was employed using microarrays. Many of these expression QTLs also show sex-specific effects on transcription. A hotspot for *trans*-acting QTLs regulating the expression of transcripts whose abundance is correlated with gonadal fat mass was identified on Chromosome 19. This region of the genome colocalizes with a clinical QTL for gonadal fat mass, suggesting that it harbors a good candidate gene for obesity.

## Introduction

Females and males share nearly identical genetic information, but vary widely with respect to disease susceptibility [1,2]. Apart from the obvious gender-specific diseases such as cervical or prostate cancer, sex influences susceptibility to nearly all highly prevalent diseases that affect both women and men, including atherosclerosis and diabetes and their precursor conditions of hyperlipidemia, obesity, and insulin resistance. These are multifactorial in their pathogenesis, encompassing environmental and behavioral aspects, as well as significant genetic determination, on which these other factors interact. The genetic component is multigenic, with the heritability embedded in the genetic variation intrinsic to our population. Although the sex differences in disease susceptibility are recognized, the interplay between sex and gene expression that is at the basis of these differences is not well understood. Females and males inherit (on average) the same genes that may be risk factors, but their expression and effect on disease risk varies significantly. An understanding and recognition of the significance of specific disease-associated polymorphisms/mutations in the context of sex is therefore of critical clinical importance.

We have utilized the mouse as a model system to study the genetics of metabolic and vascular diseases [3–5]. Rather than focus initially on natural or induced mutants of single genes, we utilize the complex endogenous genetic variation between strains in genetic crosses to identify causative genetic loci and ultimately the underlying variations responsible for trait differences [6]. This design more closely reflects the situation faced when studying human populations. The genetic composition of each individual mouse is restricted to that of the two parental strains and is defined at every locus across the genome. The application of quantitative trait locus (QTL) analysis allows the identification of those chromosomal regions that contain a genetic variation that influences trait expression (for a comprehensive review on QTLs see [7]). We [5,8], and others [9–17], have recently extended the power of this approach by incorporating genome-wide gene expression array analysis, which allows us to model the “genetics of gene expression” using similar methods. An immediate extension of this approach is toward dissecting the genetic regulation of complex phenotypes, which would greatly improve the progression from candidate locus to candidate gene.

Here we report the application of this integrated approach to study the significance of sex on the genetic determinants of obesity and the associated regulation of liver gene expression in an F2 intercross derived from the inbred strains C57BL/6J (B6) and C3H/HeJ (C3H) on an apolipoprotein E null (ApoE^−/−^) background. The BXH.ApoE^−/−^ population was designed to recapitulate several of the phenotypes associated with the so-called metabolic syndrome. The cross consists of 334 animals of both sexes, allowing us to specifically test for the dependence of QTLs on sex. We detected several thousand gene expression QTLs (eQTLs), a significant proportion of which were sex-biased. We used these analyses to dissect the genetic regulation of the gonadal fat mass trait and to identify genes associated with the trait.

## Results

### QTLs Associated with Gonadal Fat Mass

Characteristics of the B6.ApoE^−/−^ and C3H.ApoE^−/−^ parents and the F2 BXH.ApoE^−/−^ generation on a Western diet are summarized in Table 1. Gonadal fat mass differed significantly between the sexes in F2 (*p* < 10^−4^) and in the parental C3H.ApoE^−/−^ (*p* < 0.05), but not in B6.ApoE^−/−^ mice. Gonadal fat mass was the fat pad collection that represented the most animals and the most accurate collections and was thus chosen for further analysis. Broad sense heritability (*h* ^2^) calculated as (σ^2^_Total_ − σ^2^_Parental_)/σ^2^_Total_ for the gonadal fat mass trait was 54% for females and 36% for males, which is in close agreement with previous reports [18,19] and demonstrates significant heritability of gonadal fat mass.

**Table 1:**
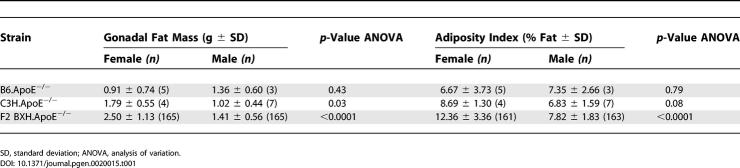
Characteristics of the BXH.ApoE^−/−^ Cross

A total of 334 F2 mice were genotyped at an average 1.5 cM density using 1,032 single nucleotide polymorphisms (SNPs) spanning all 19 autosomes. QTL analysis for several clinical traits (clinical QTLs [cQTLs]), including the unadjusted raw values for gonadal fat mass, was performed using a single marker regression approach (justified by the high-density of markers, making interval mapping unnecessary). In order to test specifically for sex effects of linkage, we included additive, dominant, sex, sex-additive, and sex-dominant parameters in our calculations (see Materials and Methods). A stepwise regression procedure was used to determine whether the addition of the final two terms significantly improved the linear regression model, conditional on realizing a significant additive QTL effect. We performed permutation analyses over all gene expression traits, estimating false discovery rates (FDRs) at different logarithm of odds (LOD) score thresholds and assessing the overall rate of QTL detection. From these analyses we constructed receiver operating characteristic (ROC)-like curves to demonstrate that our straightforward method has significantly increased power to detect QTLs compared to QTL mapping methods that do not incorporate sex and genotype–sex interactions (Figure S1). It is clear from the ROC curves that the sex and sex-interaction terms add significantly to the detection of QTL for the gene expression traits. Using previously described conventions [20], QTL models without the final two interaction terms (sex*add and sex*dom) have a suggestive threshold of 3.0 (*p* < 1 × 10^−3^) and a significant threshold of 4.3 (*p* < 5 × 10^−5^, genome-wide *p* < 0.05). QTL models incorporating only the sex*add interaction term in addition to the additive terms have one extra degree of freedom that leads to a corresponding increase in the LOD score thresholds to 3.5 (suggestive) and 4.9 (significant) for the 0.001 and 0.00005 *p*-value thresholds, respectively. QTL models incorporating both sex*add and sex*dom interaction terms possess two extra degrees of freedom, with a corresponding increase in LOD score thresholds to 4.0 (suggestive) and 5.4 (significant) for the 0.001 and 0.00005 *p*-value thresholds, respectively.

**Figure 1:**
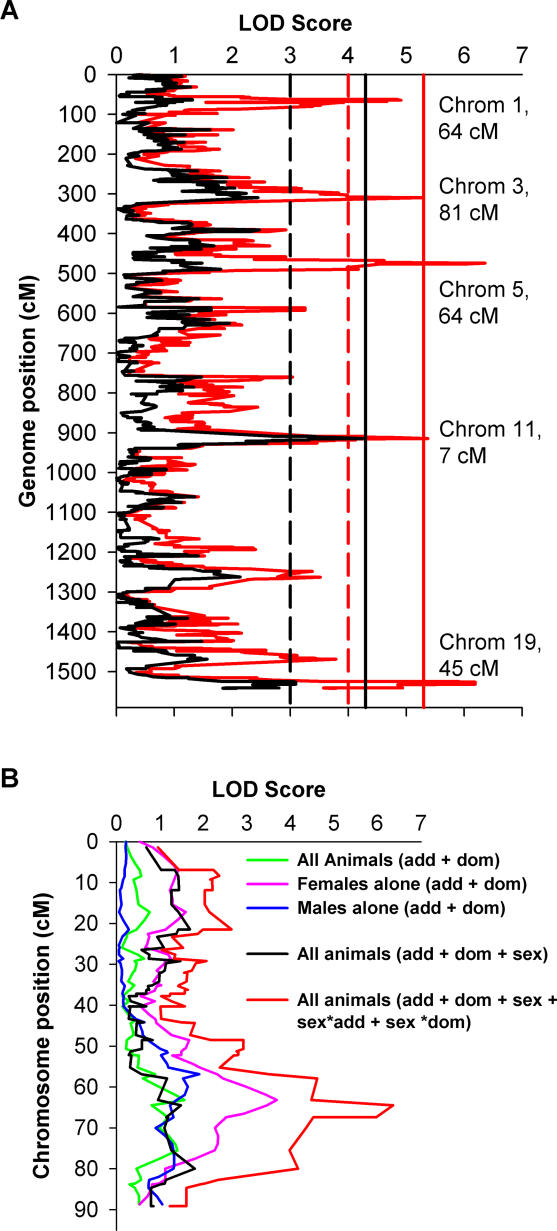
Genome Scan for Gonadal Fat Mass (A) Animals were genotyped at an average 1.5 cM density using 1,032 SNPs polymorphic between the parental strains. LOD scores computed using sex as an additive covariate (black) failed to detect significant linkage. A genome scan accounting for interactions between sex and QTL (red) showed evidence for suggestive linkage on Chromosome 1 and significant linkage on Chromosomes 3, 5, 11, and 19. Dashed and solid lines are thresholds for suggestive (*p* < 1 × 10^−3^) and significant linkage (*p* < 5 × 10^−5^), respectively. (B) Genome scans for gonadal fat mass using different models over mouse Chromosome 5. Scans for fat mass using all animals with (black) and without (green) sex as an additive covariate failed to detect significant linkage. Females analyzed alone (magenta) showed evidence for suggestive linkage (*p* < 2 × 10^−4^). When both sexes were analyzed to account for sex effects (red), a significant QTL was realized (*p* < 10^−6^). For clarity, only the model incorporating both the “sex*add” and “sex*dom” terms is shown in red, although additional models incorporating the terms separately were also computed.

One suggestive (Chromosome 1) and four significant (Chromosomes 3, 5, 11, and 19) cQTLs for the gonadal fat mass trait were identified (Figure 1A; Table 2). Four out of the five cQTLs showed statistically significant better fits with the full model incorporating the interaction terms sex*add and sex*dom, compared to the model including only the additive terms. Interestingly, the cQTL over Chromosome 11 did not improve, suggesting that the additional terms did not contribute to improved detection of this locus. Table 2 summarizes the position and LOD score of maximal linkage for each cQTL. While the focus of this study was the gonadal fat mass trait, it is noted that a genome scan for the adiposity trait resulted in cQTLs at the same locations, with very similar LOD scores and sex dependence (unpublished data).

**Table 2:**
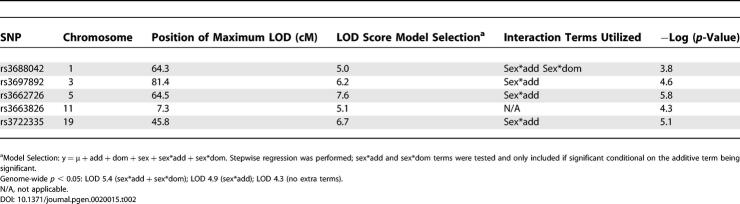
cQTLs for Gonadal Fat Mass

Results from the various regression models used to determine linkage for the Chromosome 5 cQTL are depicted in Figure 1B. Analysis of all animals with and without sex as a covariate failed to demonstrate evidence of linkage on Chromosome 5. When females were analyzed alone, a suggestive LOD score of 3.7 was realized (*p* = 2 × 10^−4^); males analyzed alone did not demonstrate evidence for linkage. However, using all 334 animals and adding the interaction terms to the QTL model significantly improved sensitivity, and a cQTL with a maximum LOD of 7.56 (*p* = 1.7 × 10^−6^) was realized.

**Figure 2:**
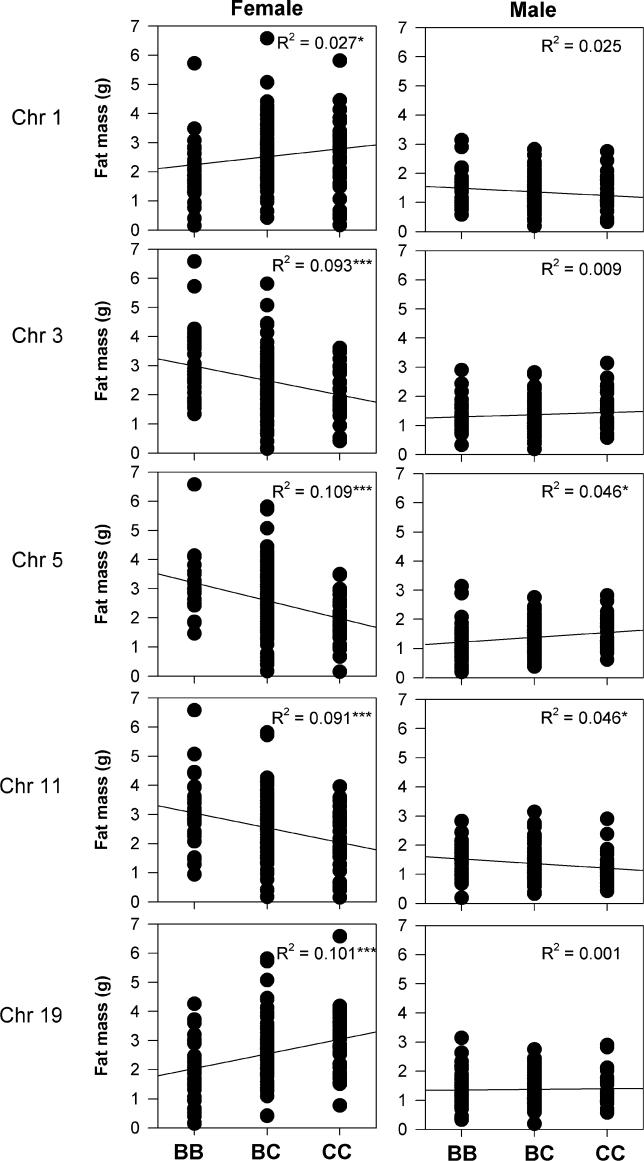
Effect of Genotype on Fat Mass Homozygous B6 (BB), C3H (CC), or heterozygous (BC) genotype at all five QTL positions, separated by sex, are shown. The underlying genotypic effects of the QTLs on fat mass differ between the sexes. Coefficients of determination (*R* ^2^) are shown along with associated ANOVAs *p < 0.05, **p < 0.01, ***p < 0.001.

Given the improved detection of four of the five cQTLs when sex-additive and sex-dominant interaction terms were considered, we hypothesized that the main genotype effect of these cQTLs on the gonadal fat mass trait would differ between the sexes (Figure 2). Indeed, cQTLs located on Chromosomes 1, 3, and 5 showed opposing effects on fat mass, or sex antagonism. The effect of the cQTL on Chromosome 11 was in the same direction in both males and females, but was sex-biased toward a larger effect in females (*R* ^2^ = 0.091 in females versus *R* ^2^ = 0.046 in males), confirming the minimal sex specificity of this cQTL. The cQTL on Chromosome 19 showed a sex-specific effect in females, with no effect in males.

Overall, all five cQTLs for gonadal fat mass were biased toward a larger effect in females. Assuming purely additive effects of each genotype, these cQTLs account for approximately 42% of the variation in female F2 mice and 13% in males, consistent with the narrow sense heritability estimates for this trait and again demonstrating significant differences in the regulation and heritability of the gonadal fat trait between the sexes.

### Liver eQTLs

**Figure 3:**
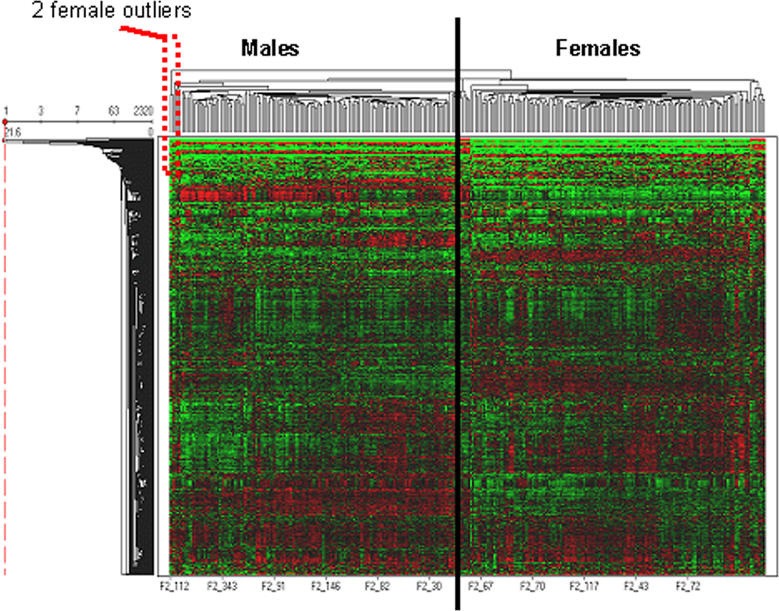
Heat Map of Liver Gene Expression Over 2,300 of the most differentially expressed genes in liver hierarchically clustered by animals (*x*-axis) against transcript levels (*y*-axis). Expression is reported as mlratio of individual experiment against a common pool. Red is over- and green underrepresented relative to pool.

Livers from 312 F2 animals (156 female, 156 male) were profiled using oligonucleotide microarrays manufactured by Agilent Technologies (Palo Alto, California, United States), which included probes for 23,574 mouse transcripts. Individual transcript intensities were corrected for experimental variation and normalized and are reported as the mean log_10_ ratio (mlratio) of an individual experiment relative to a pool composed of 150 mice randomly selected from the F2 population [21]. Each measurement was fitted to an error model and assigned a significance measurement (type I error). A heat map of the 2,320 transcripts most differentially expressed (*p* < 0.05 in 10% or more of animals) relative to the pool is depicted in Figure 3. This selection of genes was not biased on a priori known differential expression between the sexes, linkage, or correlation with a clinical phenotype. This is noteworthy because hierarchical clustering of these transcripts against the 312 F2 mice shows an almost perfect clustering into male and female subgroups, emphasizing striking effects of sex on liver gene expression levels and suggesting that sex is controlling more variance in these transcripts' expression than any other parameter.

The expression values of the 23,574 transcripts were treated as quantitative traits and fitted to the same linear regression models used to compute LOD scores for clinical traits (eQTLs). The FDR at each threshold was determined by permuting the data 100 times and taking the mean number of QTLs detected over all of the permuted datasets at a given threshold, and dividing this count by the number of QTLs detected at the same threshold in the observed data. At the threshold for significant linkage (*p* < 5 × 10^−5^, genome-wide *p* < 0.05, based on a single trait), the FDR was estimated at 3.4% for the standard QTL model not accounting for any sex terms, 3.1% for the QTL model accounting for additive sex effects, and 3.2% for the QTL model accounting for additive sex effects and allowing for the sex interaction terms to enter the model. A list of all detected suggestive (*p* < 1 × 10^−3^) and significant eQTLs (*p* < 5 × 10^−5^) detected in the BXH.ApoE^−/−^ intercross is provided in Table S1.

**Figure 4:**
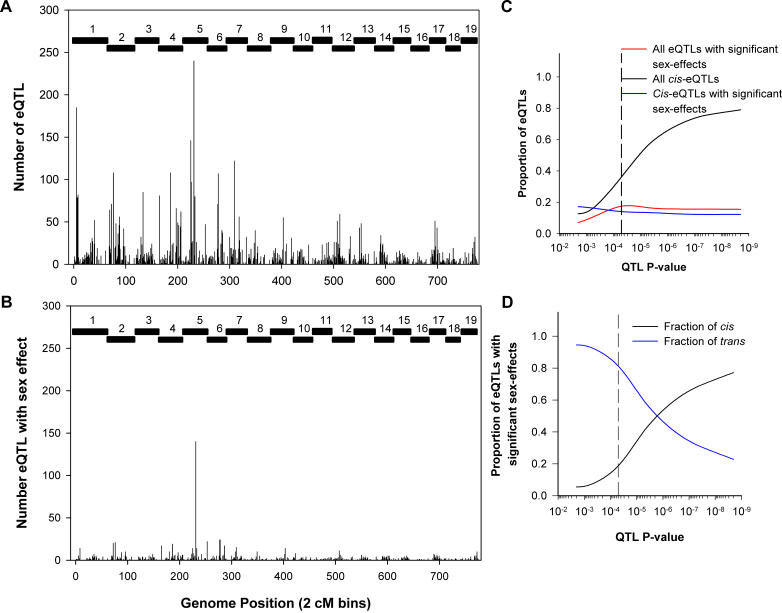
Properties of All Liver eQTLs (A) Distribution of all significant liver eQTLs across the genome in 2-cM bins. A total of 6,676 significant eQTLs were realized, representing 4,998 liver transcripts. Hotspots of nonrandom eQTL colocalization are clearly evident. (B) Distribution of eQTLs with significant sex-specific effects. A total of 1,166 eQTLs representing 1,044 transcripts show an eQTL hotspot on Chromosome 5. (C) Properties of eQTLs at increasing significance levels. As the threshold for significant linkage increases (*p*-value decreases, or LOD score increases), the proportion of *cis*-eQTLs (black) increases. The fraction of all eQTLs with sex effects (red) and *cis*-eQTLs with sex effects (blue) remains relatively constant at increasing thresholds. The dashed line indicates the genome-wide significance threshold (*p* < 5 × 10^−5^; genome-wide *p* < 0.05). (D) Properties of sex-specific eQTLs at increasing significance levels. For eQTLs with significant sex effects, as with all eQTLs, the proportion of *cis*-eQTLs (black) increases and *trans* (blue) decreases as the threshold for significance increases. At the genome-wide threshold for significance (dashed line), over 70% of eQTLs with significant sex effects are *trans.*

Characteristics of the eQTLs at different significance levels are summarized in Table 3 and shown graphically in Figure 4. We detected 6,676 eQTLs representing 4,998 transcripts at the 5 × 10^−5^ significant level. Of these, 2,118 eQTLs were located within 20 Mb (roughly 10 cM) of the corresponding gene, likely representing eQTLs regulated by *cis*-acting variation within the gene itself. Of the 6,676 significant eQTLs, 1,166 (17%) demonstrated a sex bias and were subsequently significantly improved with the addition of the sex-additive and sex-dominant terms.

**Table 3:**
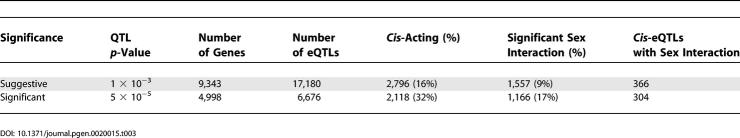
Characteristics of Liver eQTLs

The distribution of all 6,676 significant eQTLs (*p* < 5 × 10^−5^) across the genome in 2 cM bins is shown in Figure 4A. Evidence for eQTL hotspots is clear on Chromosomes 1, 2, 4, 5, 6, and 7 where significant fractions of the 6,676 eQTLs colocalize within 2 cM regions on each chromosome. Approximately 67% of eQTLs at this threshold are *trans,* and these eQTL hotspots consist primarily of *trans*-acting effects on transcriptional variation. Distribution of the 1,166 eQTLs with significant sex effects, of which 852 (73%) are *trans,* showed enrichment on Chromosome 5 at approximately 49 cM (Figure 4B) as assessed using the Fisher exact test (*p* = 8.7 × 10^−25^ after Bonferroni correction). At this locus there were 250 eQTLs, 140 of which exhibited genotype–sex interactions.

At increasing thresholds for linkage, a higher fraction of detected eQTLs were *cis*-acting (Figure 4C). The increased proportion of *cis*-eQTLs with increasing LOD score thresholds has been reported before [5,15] and confirms what is likely to be our increased power to detect first-order *cis*-acting variations affecting transcription. The proportion of eQTLs with significant sex effects remained relatively constant at all thresholds (Figure 4C). Furthermore, the majority of these sex-specific eQTLs (73%) are acting in *trans* on a given gene's expression (Figure 4D), and similar proportions of sex-specific eQTLs (26%) are *cis* compared to the proportion of all liver *cis*-eQTLs (32%). These data demonstrate the profound effects of sex on the genetic regulation of gene expression.

### *Cis*-eQTLs Are Candidate Genes for the Gonadal Fat Mass Trait

Given the marked effects of sex on liver gene expression and the genetic regulation of gonadal fat mass, we reasoned that *cis*-eQTLs with significant “sex-additive” and “sex-dominant” interactions would be potential candidate genes for our trait. Of the 2,118 significant *cis*-eQTLs, 304 (14%) were improved by the sex-interaction terms. *Cis*-eQTLs overlapping the confidence interval for the fat mass cQTL are candidate genes for the trait. Those genes with significant sex interactions receive increased consideration as potential candidates (Tables 4 and 5).

**Table 4:**
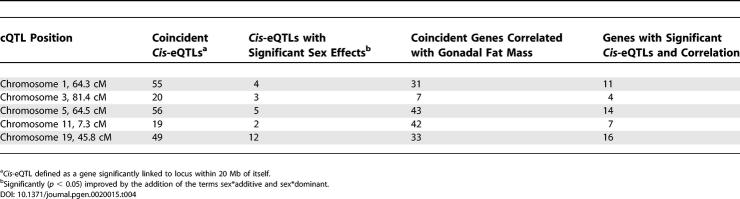
Properties of Genes Coincident with the Gonadal Fat Mass cQTL

**Table 5:**
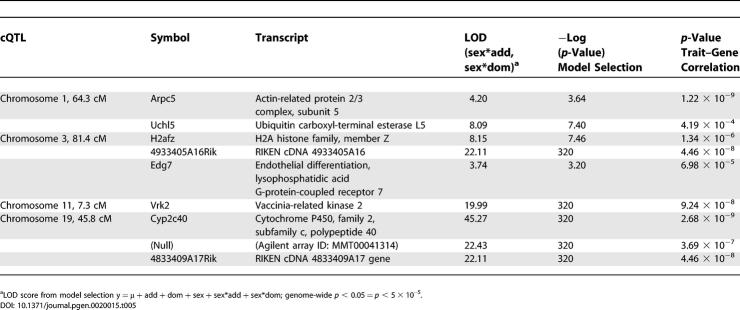
Transcripts Coincident with cQTLs with Significant Sex Effects and Correlated with Gonadal Fat Mass Are Strong Candidate Genes for the Trait

### Thousands of Genes Show a Sex-Specific Correlation with Gonadal Fat Mass

**Figure 5:**
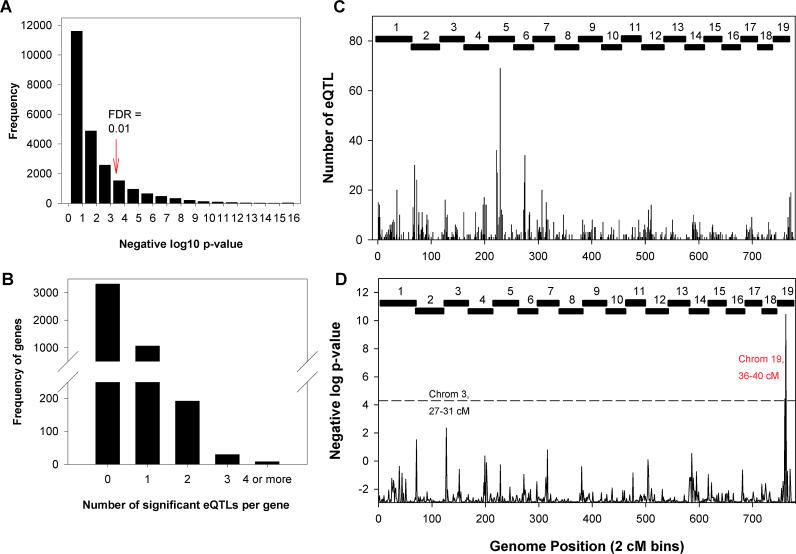
Properties of Transcripts Significantly Correlated with Gonadal Fat Mass (A) Distribution of *p*-values for trait–gene correlations between transcripts and gonadal fat mass. At FDR = 0.01, 4,613 transcripts are significantly correlated with the trait. **(**B) Number of eQTLs generated by the 4,613 genes significantly correlated with gonadal fat mass. Of these, 1,130 genes possessed at least one significant eQTL. (C) Distribution of 1,478 eQTLs significantly correlated with gonadal fat mass across the genome in 2-cM bins. (D) Identification of genomic regions enriched for eQTLs correlated with gonadal fat mass. The *x*-axis represents genome position in 2-cM bins, and the *y*-axis represents the −log_10_ Fisher exact test *p*-value for enrichment of eQTLs in overlapping 6-cM bins. The dashed line corresponds to *p* = 0.05 after correction for multiple comparisons. One significantly enriched region on Chromosome 19 is shown. The Chromosome 19 (40-cM) hotspot is coincident with a cQTL for gonadal fat mass and is highlighted in red.

For each of the 23,574 oligonucleotides represented on the array, we computed a linear regression analysis to test for association between the trait “gonadal fat mass” and each transcript abundance measure, incorporating the terms “gene,” “sex,” and “gene-by-sex,” where the “gene-by-sex” parameter tests for sex-specific correlation between a gene and the trait. As before, a stepwise regression procedure was used to determine if the addition of the interaction term significantly improved the model fit (see Materials and Methods). Multiple testing was addressed by controlling for the FDR. Distribution of the *p*-values obtained from these 23,574 correlations is shown in Figure 5A. At FDR = 0.01, 4,613 genes were significantly correlated with gonadal fat mass. Of these genes, 4,524 (98%) showed significant “gene-by-sex” effects, supporting the high degree of sex specificity in the genetic regulation of this trait. A complete list of all genes correlated with gonadal fat mass is provided in Table S2.

Of the 4,613 genes correlated with gonadal fat mass, 1,130 generate 1,478 significant eQTLs (Figure 5B). The colocalization of eQTLs for these correlated genes with the cQTL for the fat mass trait provides useful implications for the possible role of these genes. Whether the eQTLs are *cis* or *trans* determines what that role may be. Genes that show significant correlation with the gonadal fat mass trait and that have *cis*-eQTLs coincident with the fat mass cQTLs are potential candidate genes for the trait (i.e., they may contain a genetic variation in that gene that is the cause of the trait cQTL). Table 5 summarizes the genes that possess these properties for each cQTL, increasing evidence for these genes as potential candidates. As addressed below, given the complex multiorgan regulation of adipose tissue mass, it is unlikely that the genetic regulation of all five loci resides in the liver. However, some may involve the liver, and even for those that do not, the liver transcriptional variation may reflect that of the relevant tissue.

Genes that show significant correlation with gonadal fat mass and have *trans*-eQTLs coincident with the fat mass cQTL cannot be candidates directly responsible for the trait, as they are physically located elsewhere in the genome. However, they are potentially involved in the pathway(s) leading from the causative gene to the expression of the fat mass trait (i.e., their transcription is closely regulated by the causative gene at the locus). All of the five fat mass cQTLs have colocalizing *trans*-eQTLs for correlated genes. However, for a trait such as fat mass that is regulated by multiple tissues and organs, it is unlikely that all five fat mass cQTLs are primarily driven by liver gene expression. As an approach to this problem, we hypothesized that those cQTLs that are most closely associated with liver gene expression would show an overrepresentation of colocalized eQTL for correlated genes, while those loci primarily controlled by other tissues would not have shown this pattern.

To assess this, we first determined the distribution of these 1,478 eQTLs across the genome in 2-cM bins as shown in Figure 5C. In order to see if there exist any hotspots for these eQTLs, we tested eQTLs with *p* < 0.001 for enrichment along the genome in overlapping 2-cM bins against the distribution of all liver eQTLs (Figure 4A) using a Fisher exact test. Figure 5D shows the significance of enrichment reported as −log_10_ of the enrichment *p*-value across the genome. One locus on Chromosome 19 was significantly enriched for eQTLs of transcripts correlated with the gonadal fat mass trait. As anticipated, there was an overlap of a correlated eQTL hotspot and a fat mass cQTL, specifically Chromosome 19 at 40 cM. This suggests that the genetic regulation of fat mass for the Chromosome 19 locus is more closely tied to liver gene expression than are the other four fat mass cQTLs.

**Figure 6:**
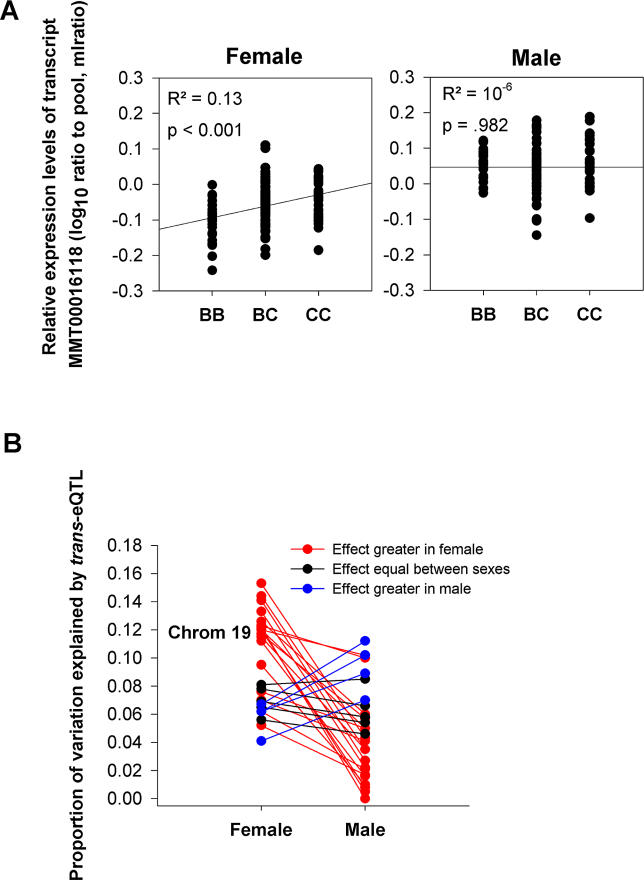
The Effects of Sex on *Trans*-eQTL Correlated with Gonadal Fat Mass (A) Example of the effect of genotype at a *trans* locus on gene expression. Presence of homozygous B6 (BB), C3H (CC), or heterozygous (BC) genotype at a *trans* locus affects transcript MMT00016118 levels (reported as mlratio) in a sex-specific manner, with effects detectable only in females. Coefficients of determination (*R* ^2^, or proportion variance explained) are shown along with associated ANOVA *p*-values. Several *trans*-eQTLs correlated with gonadal fat mass localize to regions overlapping with cQTLs for this trait, specifically, to Chromosome 19, 40 cM. (B) For Chromosome 19, the vast majority of these correlated *trans*-eQTLs are biased toward larger effects on gene expression in females (red lines). The effect of any given *trans*-eQTL is approximated as *R* ^2^ determined in a manner similar to that depicted in (A).

The effect of the *trans*-eQTLs at the Chromosome 19 locus on gene expression is summarized in Figure 6. Twenty-nine *trans*-eQTLs colocalize to Chromosome 9 at 40 cM, suggesting that 29 genes correlated with gonadal fat mass are regulated in *trans* by a polymorphism at this position. The proportion of gene expression levels controlled by this locus (approximated as the coefficients of determination *R* ^2^) differs between males and females for the majority of the transcripts (as in Figure 6A), and for Chromosome 19 (Figure 6B), females demonstrated greater genetic regulation of gene expression than males. This substantial female bias is significantly higher than would be expected to arise by chance for the Chromosome 19 locus (*p* < 0.001 by χ^2^). This locus corresponds to one of the four sex-biased cQTLs for gonadal fat mass reported in this study, and the significant sex specificity of both the *cis* and *trans* genetic regulation of liver genes correlated with fat mass supports the functional significance of this locus in this organ.

## Discussion

In this study, we described a large, densely mapped, segregating F2 mouse population designed to study the genetic regulation of several traits associated with the so-called metabolic syndrome. Several groups, including ours, have reported the advantage of combining traditional genetics with genome-wide gene expression analysis for the dissection of complex traits. This study improved on past models by including over 300 animals (three times the size of previous studies) of both sexes, allowing for the incorporation of sex-specific effects on underlying genetic regulation.

### Significant Sex Bias in the Regulation of Both Complex Traits and Gene Expression

Given the known dichotomy between females and males in the susceptibility and control of obesity, this study was designed to sufficiently power the detection of significant QTLs for this and other traits with sex-dependent effects. Note, however, that these effects can extend to traits without overall mean differences between the sexes. Previous studies have described the advantages of performing QTL analysis both with and without sex as an interactive covariate [22–25]. Analyzing the sexes separately is suboptimal since it reduces sample size in both groups, thus reducing power to detect main QTL effects, as demonstrated by our genome scan of Chromosome 5 (Figure 1B). Furthermore, separate analyses would not allow for the detection of QTLs that have opposing, or sex-antagonistic, effects in females and males and would hinder the detection of QTLs specific to one sex.

Accordingly, we detected five cQTLs for the gonadal fat mass trait on Chromosomes 1, 3, 5, 11, and 19. The detection of all five cQTLs was “driven” by the larger effect in females, with significant improvement by the incorporation of sex*additive and sex*dominant parameters. QTLs associated with obesity, gonadal fat, and abdominal fat have been reported before overlapping with cQTLs on Chromosomes 1 [26–28], 5 [26,29], and 11 [19,29] reported here, whereas the cQTL on Chromosome 3 represents a novel QTL for this trait. The Chromosome 19 cQTL for fat mass was recently reported by us [5] in the BXD intercross F2 progeny from the strains B6 and DBA (which shares the same haplotype at this region as the C3H strain used in this study). Interestingly, significant heritability and genetic regulation was seen in this F2 population despite the hyperlipidemic, proinflammatory ApoE^−/−^ background and the high-fat Western diet. This background possesses several advantages, such as allowing the modeling of human-like disease states. The predominantly female-driven effects of the five cQTLs likely reflect the significant effect of differential gonadal hormone secretions on the genetic regulation of this complex trait.

The identification of genes underlying cQTLs remains a challenge. The widespread availability of genome-wide expression analysis has begun to address this by providing a snapshot of transcription in relevant organs and thus providing initial information for which genes can differentiate a given trait. Furthermore, by treating transcript levels as quantitative traits, we can map the genetic regulation underlying differential gene expression (eQTLs). Those eQTLs that have *cis*-acting variations affecting their transcription are potential candidate genes for the trait. At a single trait, genome-wide significance level of 0.05, we detected 6,676 eQTLs representing 4,998 genes, of which 2,118 were *cis*-acting. At increased thresholds, the proportion of *cis*-eQTLs increased, which is in good agreement with previous studies [5,15] and likely reflects the increased power to detect *cis*-acting variations affecting transcription. Of all 6,676 significant eQTLs, 1,166 possessed significant sex interactions. Of these, 304 were *cis* and 852 were *trans,* suggesting that only a minority of the sex-specific effects on the regulation of gene expression occur through polymorphisms within the gene itself. Rather, underlying genetic regulation of most transcripts is the result of interactions between *trans* loci and sex-specific factors (e.g., hormones). As with cQTLs, sex bias in the predominantly *trans* genetic regulation of gene expression is likely secondary to different sex hormone profiles.

Recently, using a similar dataset, our group demonstrated that significant *cis*-eQTLs (*p* < 5 × 10^−5^) largely represent true positives [30] and are enriched for highly polymorphic regions over the mouse genome. The *cis*-eQTLs presented in Table 5 overlap with one of the gonadal fat mass cQTLs and should be considered potential candidates. Given the sex effects in the gonadal fat mass cQTLs, we reasoned that the *cis*-eQTLs with significant sex*additive and sex*dominant effects should receive priority consideration. The use of eQTLs to dissect cQTLs is a method still in its infancy, with uncertain efficacy and applicability. Nevertheless, application of this analysis to this dataset provides some tantalizingly attractive candidate genes.

One shortcoming of this approach, however, is that candidate genes are limited to those whose transcript expression levels vary in association with a nearby polymorphism that differs between the parental strains—in other words, genes with significant and detectable *cis*-eQTLs. However, it is not strictly necessary for candidate genes to have evidence of such linkage: polymorphisms underlying a trait cQTL can affect gene function or posttranslational modifications. Nevertheless, several phenotypes are known to be regulated, at least partly, at the level of transcription or mRNA stability, which is exactly what our methods are designed to detect. A separate problem is that organ-specific gene expression differences may preclude one from detecting the relevant causative gene if the tissue arrayed is not the tissue where the control is exerted. This is particularly relevant for a trait such as adipose tissue mass, which is controlled by multiple tissues. We propose that analysis of correlated genes can provide guidance as discussed below.

### Genes Correlated with Gonadal Fat Mass Illustrate Tissue-Specific Regulation of the Trait

In an effort to identify genes associated with the fat mass trait, but not necessarily *candidate* genes underlying the trait cQTLs, we fitted linear models to assess the degree of association between transcripts and gonadal fat mass. As with QTLs, sex-specific correlations were modeled. At an FDR of 1%, 4,613 genes were found to be significantly correlated with gonadal fat mass, of which 4,254 (98%) showed sex-biased correlation. As indicated in Tables 4 and 5, several genes with detectable *cis*-eQTLs are also significantly correlated with the trait and are even further prioritized as candidate genes.

Thus far, studies that have examined the “genetics of gene expression” are in good agreement regarding the increased power to detect *cis*-eQTLs relative to *trans* [5,9,15,16,31]. It is unclear at this time, however, what exactly is the significance of *trans*-eQTLs and the nature of the underlying polymorphisms associated with them. Furthermore, the eQTL hotspots reported in this and previous studies [5,9,10] largely represent *trans*-eQTLs. This localization suggests some functional significance to these regions. Of the 4,613 genes correlated with gonadal fat mass, 1,130 generate 1,478 significant eQTLs, of which 1,023 (69%) are *trans*-acting. These eQTLs are significantly enriched at one locus (Chromosome 19). Interestingly, this hotspot was coincident with a cQTL associated with fat mass reported in this study. Since these transcripts represent those significantly correlated with gonadal fat mass, the localization of their eQTLs to these regions strongly supports the notion that the genes with *trans*-eQTLs represent downstream targets of candidate regulatory genes located at the position of significant linkage. This means that the genes may be causal but downstream of the gene responsible for the cQTLs, or they may be reacting to the increased gonadal fat mass and associated metabolic changes. These data also suggest that identifying such loci that show overrepresentation of highly correlated genes is a means to identify which of the trait cQTLs are more likely controlled by the tissue arrayed. As expected, the Chromosome 19 locus was enriched for *trans*-eQTLs with substantially greater effects in females. Functional and promoter analysis of genes with a common *trans*-eQTL may prove enlightening. Furthermore, gene expression network construction and analysis may be improved by the incorporation of experimentally demonstrated *cis* versus *trans* regulation.

### Conclusion

The integration of traditional genetics with genome-wide expression analysis was first proposed by Jansen and Nap 4 y ago [32]. Advances in genomic technology and bioinformatic resources since then have vastly improved the applicability of these methods to the dissection of complex traits. Taking into account sex-specific effects will similarly improve the sensitivity to detect underlying genetic regulation, especially for phenotypes known to be affected by sex. Furthermore, network analyses are increasingly being applied to complex phenotypes [11,33]. Regardless of which variables are used in the construction of these networks, whether they measure gene expression or protein interactions, accounting for sex specificity, hormonal status, or construction of different networks for females and males would likely more accurately represent the complexity associated with these phenotypes.

We reported here on the initial genetic and genomic analysis of an F2 intercross population designed to recapitulate several traits associated with human metabolic syndrome. Using 334 mice of both sexes genotyped at high density, this is the largest study of its kind to date designed, and it is strongly powered to detect subtle effects of genetic regulation and sex specificity. We identified five cQTLs for the gonadal fat mass trait, all with greater effects in females. We also detected several thousand significant liver eQTLs, a significant fraction of which are sex-biased, demonstrating how meaningful effects of sex on gene expression extend beyond overall mean differences. We demonstrated the application of linkage and correlation methods to identify candidate genes. Finally, we showed that localization of a subset of liver genes linked in *trans* to a cQTL region can identify relative tissue contributions to the genetic regulation of a complex trait. We anticipate that the application of these and similar methods would significantly improve the elucidation of the genetic regulation underlying complex phenotypes.

## Materials and Methods

### Animals and tissue collection.

C57BL/6J ApoE^−/−^ (B6.ApoE^−/−^) were purchased from Jackson Laboratory (Bar Harbor, Maine, United States). C3H/HeJ ApoE^−/−^ (C3H.ApoE^−/−^) were generated by backcrossing B6.ApoE^−/−^ to C3H for ten generations. F1 mice were generated from reciprocal intercrossing between B6.ApoE^−/−^ and C3H.ApoE^−/−^, and F2 mice were subsequently bred by intercrossing F1 mice. A total of 334 mice (169 female, 165 male) were produced. All mice were fed Purina Chow containing 4% fat until 8 wk of age and then transferred to a “Western” diet containing 42% fat and 0.15% cholesterol for 16 wk. Mice were sacrificed at 24 wk of age. At death, livers were immediately collected and flash-frozen in liquid N_2_, and gonadal fat pads were extracted and weighed.

### RNA sample preparation, microarray hybridization, and expression analysis.

RNA preparation and array hybridizations were performed at Rosetta Inpharmatics (Seattle, Washington, United States). The custom ink-jet microarrays used in this study (Agilent Technologies, previously described [21,34]) contain 2,186 control probes and 23,574 noncontrol oligonucleotides extracted from mouse UniGene clusters and combined with RefSeq sequences and RIKEN full-length clones.

Mouse livers were homogenized and total RNA extracted using TRIzol reagent (Invitrogen, Carlsbad, California, United States) according to manufacturer's protocol. Three micrograms of total RNA was reverse transcribed and labeled with either Cy3 or Cy5 fluorochrome. Purified Cy3 or Cy5 complementary RNA was hybridized to at least two microarray slides with fluor reversal for 24 h in a hybridization chamber, washed, and scanned using a laser confocal scanner. Arrays were quantified on the basis of spot intensity relative to background, adjusted for experimental variation between arrays using average intensity over multiple channels, and fitted to an error model to determine significance (type I error). Gene expression is reported as the mlratio relative to the pool derived from 150 mice randomly selected from the F2 population. For subsequent analyses, mlratio data are assumed to be normally distributed, a valid assumption as previously demonstrated [21,30]. The error model used to assess whether a given gene is significantly differentially expressed in a single sample relative to a pool comprised of a randomly selected subset of 150 samples has been extensively described and tested in a number of publications [35,36].

### Genotyping and linkage statistics.

Genomic DNA was isolated from kidney by phenol-chloroform extraction. An examination of existing databases identified over 1,300 SNPs that showed variation between the B6 and C3H strains, and a complete linkage map for all 19 autosomes was constructed using 1,032 of these SNPs at an average density of 1.5 cM. Genotyping was conducted by ParAllele (South San Francisco, California, United States) using the molecular-inversion probe (MIB) multiplex technique [37]. Testing for linkage of both clinical traits and gene expression (using mlratio) was conducted using a linear model. Consider a phenotype denoted by *y.* The linear model that relates variation in *y* to QTLs and other covariates (e.g., sex) is given by the general form


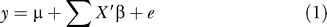


where μ is the trait mean, X′ a vector of covariates, β being the associated vector of regression coefficients, and *e* the residual error. Linkage was computed for over 20 clinical traits as well as 23,574 liver transcripts. Standard genome scans calculate linkage by comparing the linear model


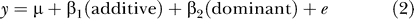


to the null model


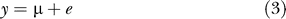


where β_1_ and β_2_ are the regression coefficients of the additive and dominant parameters, respectively. The LOD score represents the difference in the log_10_ of the likelihood of the above two equations, where the individual model likelihoods are maximized with respect to the model parameters, given the marker genotype and phenotype data. If a trait *y* differs on average between the two sexes but the QTL has the same effect in both males and females, we can model this interaction by including sex as an additive covariate in the above models, resulting in the new model


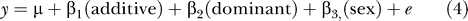


which is then compared to the null model


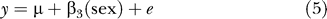


where β_3_ is the regression coefficient of the sex parameter. The effect of a QTL may be dependent on the state of a covariate; for instance, a QTL may have an effect specific to one sex, or may have opposite effects in the two sexes. This interaction can be modeled using a full model, which accounts for all additive covariates, as well as interactions between the covariates:


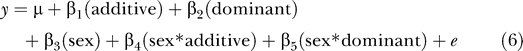


which is compared to the above null model (Equation 5).

The full model (Equation 6) allows us to model all heritable and sex-specific interactions in a single equation and maximally powers us to detect significant QTLs when the sex and sex-interaction terms are significant, given all 334 animals are included in the analysis. Furthermore, using the full model obviates the need for modeling QTLs in one sex only, a procedure that could decrease our power by halving the sample size, rendering it impossible to detect interactions between QTLs and sex. However, because the full model contains two more parameters than the model that treats sex as an additive covariate (Equation 4), the LOD score threshold for significant linkage is higher (using the convention of Lander and Kruglyak [20]), with QTL-specific significance levels of 2 × 10^−3^ and 5 × 10^−5^ equivalent to LOD scores of 4.0 (suggestive linkage) and 5.4 (significant linkage, equivalent to genome-wide *p* < 0.05), respectively. As a result, if there are no significant sex-additive or sex-dominant interactions, the full model will actually reduce power to detect linkage. To minimize the loss in power of fitting the full model when the sex-interaction terms are not significant, we employed a model selection procedure that introduces sex-interaction terms only if they add significantly to the overall QTL model.

The model selection procedure makes use of forward stepwise regression techniques to determine whether it is beneficial to include the sex-interaction terms, conditional on realizing a significant additive effect (*p* < 0.001). That is, the data are fitted to Equation 4 for a given marker, and if the add term is significant at the 0.001 significance level, then we attempt to add the sex*add term into the model. The sex*add term is retained in the model if the Bayesian information criterion (BIC) for this model is smaller than the BIC for Equation 4. If sex*add is included in the model as a result of this procedure, then we again use BIC to consider including the sex*dom term in the model.

To determine which of the four models (Equations 2, 4, 6, and the model selection) is optimal, we empirically estimated the FDR for each model over a set of 3,000 genes randomly selected from the set of all genes detected as expressed in the liver samples. For each gene and for each marker we fitted each of the four models to the data. We performed this same analysis on ten separate permutation sets in which each of the 3,000 gene expression trait vectors was permuted such that the correlation structure among the genes was preserved. The FDR for a given LOD score threshold was then computed as the ratio of the mean number of QTL detected in the permuted datasets (the mean taken over the ten permuted datasets) and the total number of QTL detected in the observed 3,000-gene dataset. We then generated ROC-like curves by varying the LOD score threshold, resulting in a range of FDRs (from 0% to more than 50%). To simplify this type of summary, we considered no more than one QTL per chromosome per gene expression trait by considering only the QTL with the max LOD score on each chromosome for each trait. The ROC curves for each of the four models are shown in Figure S1, with the black curve corresponding to Equation 2, the blue curve corresponding to Equation 4, the red curve corresponding to Equation 6, and the green curve corresponding to the model selection procedure.

It is clear from the ROC curves that the sex and sex-interaction terms are significant in the gene expression dataset. For example, at an FDR of 5% we note that 800 QTLs were detected in the set of 3,000 genes for model 1, while 968 (21% increase) and 1,159 (45% increase) QTLs were detected for Equations 4 and 6, respectively. These results demonstrate significantly increased power to detect QTLs when sex is taken into account. We further note that the stepwise selection procedure captures more information than Equation 4, indicating a significant interaction signature in this dataset and also demonstrating that this simple statistical procedure is capable of identifying significant interaction events even at conservative FDR thresholds. Finally, it is of particular note that Equation 4 performed better than Equation 6, the model that incorporated the interaction terms at all times. That is, despite there being a significant interaction signature, the signature was not large enough to justify including interaction terms for every expression trait and at every marker tested. This fact motivated the need to employ the forward regression procedure, and these results further motivate the need to explore sex effects by employing even more sophisticated QTL detection methods, such as that recently described by Yi et al. [38].

### Additional statistics.

For each 23,574 oligonucleotides represented on the array, we computed a linear regression analysis to test for a correlation between the trait “gonadal fat mass” and each transcript. Similar to our method of calculating linkage, we employed a stepwise linear regression procedure using equations of the form


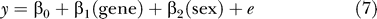


or


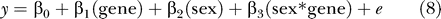


compared to the null model


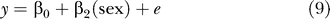


where β_0_ is the intercept, and β_1_, β_2_, and β_3_ represent the regression coefficients of their respective terms. As before, the parameter “sex*gene” is only retained if it significantly improves the fit of the model. The *p*-value threshold for significant correlation is calculated by an F test, which compares the appropriate model (Equation 7 or 8) to the null model (Equation 9). As before, multiple testing was addressed with use of the FDR, ranking the *p*-values obtained from the above F tests and setting α = 0.01.

Genes correlated with the gonadal fat mass trait generated several significant eQTLs. In order to determine if eQTLs generated by these genes were enriched in any locus or if they were distributed randomly, we compared the distribution of these eQTLs against the distribution of all liver eQTLs in overlapping 6-cM bins across the genome using the Fisher exact test. This test is based on exact probabilities from a specific distribution (hypergeometric distribution). *p*-Values obtained from this test were corrected for multiple comparisons using a simple Bonferonni correction (given that we performed 772 tests across the genome, 0.05/772). Loci with *p* < 6.5 × 10^−5^ by Fisher exact test were considered significantly enriched for eQTLs correlated with gonadal fat mass.

## Supporting Information

Figure S1 ROC Curves for Each of the Four Models (21 KB DOC)

Table S1 Suggestive and Significant eQTLs (3.7 MB XLS)

Table S2 Transcripts Significantly Correlated with Gonadal Fat Mass (716 KB XLS)

## Abbreviations

ApoE^−/−^: apolipoprotein E null
cQTL: clinical QTL
eQTL: gene expression QTL
FDR: false discovery rate
LOD: logarithm of odds
mlratio: mean log_10_ ratio
QTL: quantitative trait locus
ROC: receiver operating characteristic
SNP: single nucleotide polymorphism

## References

[pgen-0020015-b001] Rossouw JE (2002). Hormones, genetic factors, and gender differences in cardiovascular disease. Cardiovasc Res.

[pgen-0020015-b002] Lusis AJ (2003). Genetic factors in cardiovascular disease: 10 questions. Trends Cardiovasc Med.

[pgen-0020015-b003] Drake TA, Schadt E, Hannani K, Kabo JM, Krass K (2001). Genetic loci determining bone density in mice with diet-induced atherosclerosis. Physiol Genomics.

[pgen-0020015-b004] Colinayo VV, Qiao JH, Wang X, Krass KL, Schadt E (2003). Genetic loci for diet-induced atherosclerotic lesions and plasma lipids in mice. Mamm Genome.

[pgen-0020015-b005] Schadt EE, Monks SA, Drake TA, Lusis AJ, Che N (2003). Genetics of gene expression surveyed in maize, mouse and man. Nature.

[pgen-0020015-b006] Churchill GA, Airey DC, Allayee H, Angel JM, Attie AD (2004). The collaborative cross: A community resource for the genetic analysis of complex traits. Nat Genet.

[pgen-0020015-b007] Abiola O, Angel JM, Avner P, Bachmanov AA, Belknap JK (2003). The nature and identification of quantitative trait loci: A community's view. Nat Rev Genet.

[pgen-0020015-b008] Monks SA, Leonardson A, Zhu H, Cundiff P, Pietrusiak P (2004). Genetic inheritance of gene expression in human cell lines. Am J Hum Genet.

[pgen-0020015-b009] Brem RB, Yvert G, Clinton R, Kruglyak L (2002). Genetic dissection of transcriptional regulation in budding yeast. Science.

[pgen-0020015-b010] Yvert G, Brem RB, Whittle J, Akey JM, Foss E (2003). Trans-acting regulatory variation in Saccharomyces cerevisiae and the role of transcription factors. Nat Genet.

[pgen-0020015-b011] Kirst M, Myburg AA, De Leon JP, Kirst ME, Scott J (2004). Coordinated genetic regulation of growth and lignin revealed by quantitative trait locus analysis of cDNA microarray data in an interspecific backcross of eucalyptus. Plant Physiol.

[pgen-0020015-b012] Kirst M, Basten CJ, Myburg AA, Zeng ZB, Sederoff RR (2005). Genetic architecture of transcript level variation in differentiating xylem of an eucalyptus hybrid. Genetics.

[pgen-0020015-b013] Bystrykh L, Weersing E, Dontje B, Sutton S, Pletcher MT (2005). Uncovering regulatory pathways that affect hematopoietic stem cell function using “genetical genomics.”. Nat Genet.

[pgen-0020015-b014] Chesler EJ, Lu L, Shou S, Qu Y, Gu J (2005). Complex trait analysis of gene expression uncovers polygenic and pleiotropic networks that modulate nervous system function. Nat Genet.

[pgen-0020015-b015] Hubner N, Wallace CA, Zimdahl H, Petretto E, Schulz H (2005). Integrated transcriptional profiling and linkage analysis for identification of genes underlying disease. Nat Genet.

[pgen-0020015-b016] Morley M, Molony CM, Weber TM, Devlin JL, Ewens KG (2004). Genetic analysis of genome-wide variation in human gene expression. Nature.

[pgen-0020015-b017] Wayne ML, McIntyre LM (2002). Combining mapping and arraying: An approach to candidate gene identification. Proc Natl Acad Sci U S A.

[pgen-0020015-b018] West DB (1996). Genetics of obesity in humans and animal models. Endocrinol Metab Clin North Am.

[pgen-0020015-b019] Brockmann GA, Haley CS, Renne U, Knott SA, Schwerin M (1998). Quantitative trait loci affecting body weight and fatness from a mouse line selected for extreme high growth. Genetics.

[pgen-0020015-b020] Lander E, Kruglyak L (1995). Genetic dissection of complex traits: Guidelines for interpreting and reporting linkage results. Nat Genet.

[pgen-0020015-b021] He YD, Dai H, Schadt EE, Cavet G, Edwards SW (2003). Microarray standard data set and figures of merit for comparing data processing methods and experiment designs. Bioinformatics.

[pgen-0020015-b022] Stone JL, Merriman B, Cantor RM, Yonan AL, Gilliam TC (2004). Evidence for sex-specific risk alleles in autism spectrum disorder. Am J Hum Genet.

[pgen-0020015-b023] Solberg LC, Baum AE, Ahmadiyeh N, Shimomura K, Li R (2004). Sex- and lineage-specific inheritance of depression-like behavior in the rat. Mamm Genome.

[pgen-0020015-b024] Korstanje R, Li R, Howard T, Kelmenson P, Marshall J (2004). Influence of sex and diet on quantitative trait loci for HDL cholesterol levels in an SM/J by NZB/BlNJ intercross population. J Lipid Res.

[pgen-0020015-b025] Corbett J, Saccone NL, Foroud T, Goate A, Edenberg H (2005). A sex-adjusted and age-adjusted genome screen for nested alcohol dependence diagnoses. Psychiatr Genet.

[pgen-0020015-b026] Keightley PD, Hardge T, May L, Bulfield G (1996). A genetic map of quantitative trait loci for body weight in the mouse. Genetics.

[pgen-0020015-b027] Moody DE, Pomp D, Nielsen MK, Van Vleck LD (1999). Identification of quantitative trait loci influencing traits related to energy balance in selection and inbred lines of mice. Genetics.

[pgen-0020015-b028] Zhang S, Gershenfeld HK (2003). Genetic contributions to body weight in mice: Relationship of exploratory behavior to weight. Obes Res.

[pgen-0020015-b029] Snyder EE, Walts B, Perusse L, Chagnon YC, Weisnagel SJ (2004). The human obesity gene map: The 2003 update. Obes Res.

[pgen-0020015-b030] Doss S, Schadt EE, Drake TA, Lusis AJ (2005). *Cis*-acting expression quantitative trait loci in mice. Genome Res.

[pgen-0020015-b031] Carlborg O, De Koning DJ, Manly KF, Chesler E, Williams RW (2005). Methodological aspects of the genetic dissection of gene expression. Bioinformatics.

[pgen-0020015-b032] Jansen RC, Nap JP (2001). Genetical genomics: The added value from segregation. Trends Genet.

[pgen-0020015-b033] Zhu J, Lum PY, Lamb J, GuhaThakurta D, Edwards SW (2004). An integrative genomics approach to the reconstruction of gene networks in segregating populations. Cytogenet Genome Res.

[pgen-0020015-b034] Schadt EE, Li C, Su C, Wong WH (2000). Analyzing high-density oligonucleotide gene expression array data. J Cell Biochem.

[pgen-0020015-b035] Roberts CJ, Nelson B, Marton MJ, Stoughton R, Meyer MR (2000). Signaling and circuitry of multiple MAPK pathways revealed by a matrix of global gene expression profiles. Science.

[pgen-0020015-b036] Hughes TR, Marton MJ, Jones AR, Roberts CJ, Stoughton R (2000). Functional discovery via a compendium of expression profiles. Cell.

[pgen-0020015-b037] Hardenbol P, Yu F, Belmont J, Mackenzie J, Bruckner C (2005). Highly multiplexed molecular inversion probe genotyping: Over 10,000 targeted SNPs genotyped in a single tube assay. Genome Res.

[pgen-0020015-b038] Yi N, Yandel BS, Churchill GA, Allison DB, Eisen EJ (2005). Bayesian model selection for genome-wide epistatic quantitative trait loci analysis. Genetics.

