# Supplementary material for: Genetic and Genomic Analysis of a Fat Mass Trait with Complex Inheritance Reveals Marked Sex Specificity
Source: PLoS Genet. 2006 Feb 3;2(2):e15. doi: 10.1371/journal.pgen.0020015 (PMC1359071; doi:10.1371/journal.pgen.0020015)
Supplement: Supplementary file 1 [file pgen.0020015.sg001.doc]

Supplementary Figure 1

To determine the model that best fits the data for a single marker regression approach, we compared 4 different models:

1.
2.
3.
4. Model selection procedure

The data were fit to each of these models for each gene expression trait at each marker. Permutations were performed on a random selection of 3000 gene expression traits and also fit to each model. The FDR at a given LOD score for each model was computed as the ratio of the mean number of QTL detected in the permuted data sets (the mean taken over the 10 permuted data sets) and the total number of QTL detected in the observed 3000-gene data set. We then generated receiver operating characteristic curves by varying the LOD score threshold, resulting in a range FDRs (from 0 to more than 50%). To simplify this type of summary, we considered no more than one QTL per chromosome per gene expression trait by considering only the QTL with the max LOD score on each chromosome for each trait. The ROC curves for each of the four models are shown above, with the black curve corresponding to model 1, the blue curve corresponding to model 2, the red curve corresponding to model 3, and the green curve corresponding to the model selection procedure.
